# 2′-deoxy-ADPR activates human TRPM2 faster than ADPR and thereby induces higher currents at physiological Ca^2+^ concentrations

**DOI:** 10.3389/fimmu.2024.1294357

**Published:** 2024-01-22

**Authors:** Jelena Pick, Simon Sander, Stefanie Etzold, Anette Rosche, Henning Tidow, Andreas H. Guse, Ralf Fliegert

**Affiliations:** ^1^ The Calcium Signaling Group, Department of Biochemistry and Molecular Cell Biology, University Medical Center Hamburg-Eppendorf, Hamburg, Germany; ^2^ The Hamburg Advanced Research Center for Bioorganic Chemistry (HARBOR) & Department of Chemistry, Institute for Biochemistry and Molecular Biology, University of Hamburg, Hamburg, Germany

**Keywords:** TRPM2, ADPr, 2′-deoxy-ADPR, calcium signaling, calcium sensitivity, calmodulin

## Abstract

TRPM2 is a Ca^2+^ permeable, non-selective cation channel in the plasma membrane that is involved in the innate immune response regulating, for example, chemotaxis in neutrophils and cytokine secretion in monocytes and macrophages. The intracellular adenine nucleotides ADP-ribose (ADPR) and 2′-deoxy-ADPR (2dADPR) activate the channel, in combination with their co-agonist Ca^2+^. Interestingly, activation of human TRPM2 (hsTRPM2) by 2dADPR is much more effective than activation by ADPR. However, the underlying mechanism of the nucleotides’ differential effect on the channel is not yet fully understood. In this study, we performed whole-cell patch clamp experiments with HEK293 cells heterologously expressing hsTRPM2. We show that 2dADPR has an approx. 4-fold higher Ca^2+^ sensitivity than ADPR (EC_50_ = 190 and 690 nM). This allows 2dADPR to activate the channel at lower and thus physiological intracellular Ca^2+^ concentrations. Kinetic analysis of our data reveals that activation by 2dADPR is faster than activation by ADPR. Mutation in a calmodulin binding N-terminal IQ-like motif in hsTRPM2 completely abrogated channel activation by both agonists. However, mutation of a single amino acid residue (W1355A) in the C-terminus of hsTRPM2, at a site of extensive inter-domain interaction, resulted in slower activation by 2dADPR and neutralized the difference in rate of activation between the two agonists. Taken together, we propose a mechanism by which 2dADPR induces higher hsTRPM2 currents than ADPR by means of faster channel activation. The finding that 2dADPR has a higher Ca^2+^ sensitivity than ADPR may indicate that 2dADPR rather than ADPR activates hsTRPM2 in physiological contexts such as the innate immune response.

## 1 Introduction

TRPM2 belongs to the melastatin subfamily of TRP ion channels. It is a non-selective cation channel that is both permeable for and activated by Ca^2+^ (1). Being a Ca^2+^ channel, TRPM2 plays a role in diverse cellular processes such as oxidative stress-induced cell death (2), sensation of warmth (3, 4) and insulin secretion (5). It is also expressed in many cells of the innate immune system (6) and regulates chemotaxis (7, 8) and cytokine secretion (9, 10), among others. Its role in these different cell functions links TRPM2 to a variety of pathologies, for example Alzheimer’s disease (11), obesity (12), type 2 diabetes (12) and multiple sclerosis (13). It is therefore highly desirable to further understand the complex activation mechanism of TRPM2, including the interplay between its co-agonists Ca^2+^ and ADP-ribose (ADPR) or 2′-deoxy-ADPR (2dADPR).

Human TRPM2 (hsTRPM2) forms homotetramers. Each monomer/subunit is composed of the N-terminal melastatin homology regions (MHR) 1-4, six transmembrane helices, the TRP helices 1-2, Rib and Pole helices and the characteristic C-terminal NUDT9 homology (NUDT9H) domain (14, 15). The fact that this domain is homologous to the ADPR pyrophosphatase NUDT9 brought on the discovery of ADPR as an agonist of TRPM2 (1). However, the activation of TRPM2 additionally requires Ca^2+^. Ca^2+^ activates the channel from the intracellular side, but once the channel is open, extracellular Ca^2+^ provides positive feedback (16–18). A cryo-EM structure of hsTRPM2 suggests that upon activation ADPR binds to the MHR1/2 and NUDT9H domain, whereas Ca^2+^ binds to a site that is formed by residues of transmembrane helices 2, 3 and TRP helix 1 and is conserved in TRPM2 from other species (15). Mutation of this site indeed reduces Ca^2+^ sensitivity in sea anemone (*nv*) TRPM2 (19). All Ca^2+^ sensitive TRPM channels share this Ca^2+^ binding site (20), but it is known for some of them, for example for TRPM4, that the regulation of Ca^2+^ sensitivity is multifactorial (21–24). Consistently, calmodulin (17, 25, 26), an N-terminal EF-hand (27) and phosphatidylinositol 4,5-bisphosphate (PIP_2_) (19, 28, 29) are suspected to also facilitate the Ca^2+^ sensitivity of TRPM2. Concerning calmodulin, Tong et al. (25) discovered an N-terminal IQ-like motif (aa 406-416) and showed that mutation of this motif abolishes the intracellular Ca^2+^ signal after TRPM2 activation. Yet, it remains unclear how these different factors/binding sites individually and quantitatively contribute to the Ca^2+^ sensitivity of TRPM2.

While searching for an ADPR analog that inhibits TRPM2, Fliegert et al. (30) discovered that activation of hsTRPM2 by 2dADPR is much more effective than activation by ADPR. They proposed a potential biosynthetic pathway and detected endogenous 2dADPR in Jurkat T cells. The observed increase in endogenous 2dADPR after stimulation would be consistent with 2dADPR acting as an endogenous superagonist of hsTRPM2. Experiments at selected intracellular Ca^2+^ concentrations suggested that activation of hsTRPM2 by 2dADPR has a different Ca^2+^ sensitivity than activation by ADPR. Interestingly, mutation of a C-terminal calmodulin binding motif (aa 1355-1368), which plays a role in the regulation of hsTRPM2 by temperature, resulted in similar whole-cell currents induced by 2dADPR and ADPR (31). Further, it was recently shown that both nucleotide binding sites in the MHR1/2 and NUDT9H domain are necessary for the activation of hsTRPM2 by 2dADPR (32), as it is the case for ADPR (15). However, full characterization of the Ca^2+^ sensitivity of the nucleotide agonist’s activity is still missing.

Accordingly, in this study, we analyzed activation of hsTRPM2 by 2dADPR and ADPR at different intracellular Ca^2+^ concentrations. Further, we investigated any role of the two known calmodulin binding sites in hsTRPM2 activation. Finally, we propose a mechanism by which 2dADPR induces higher whole-cell currents than ADPR in hsTRPM2.

## 2 Results

### 2.1 Increased Ca^2+^ sensitivity of hsTRPM2 activation by 2dADPR

**Figure 1:**
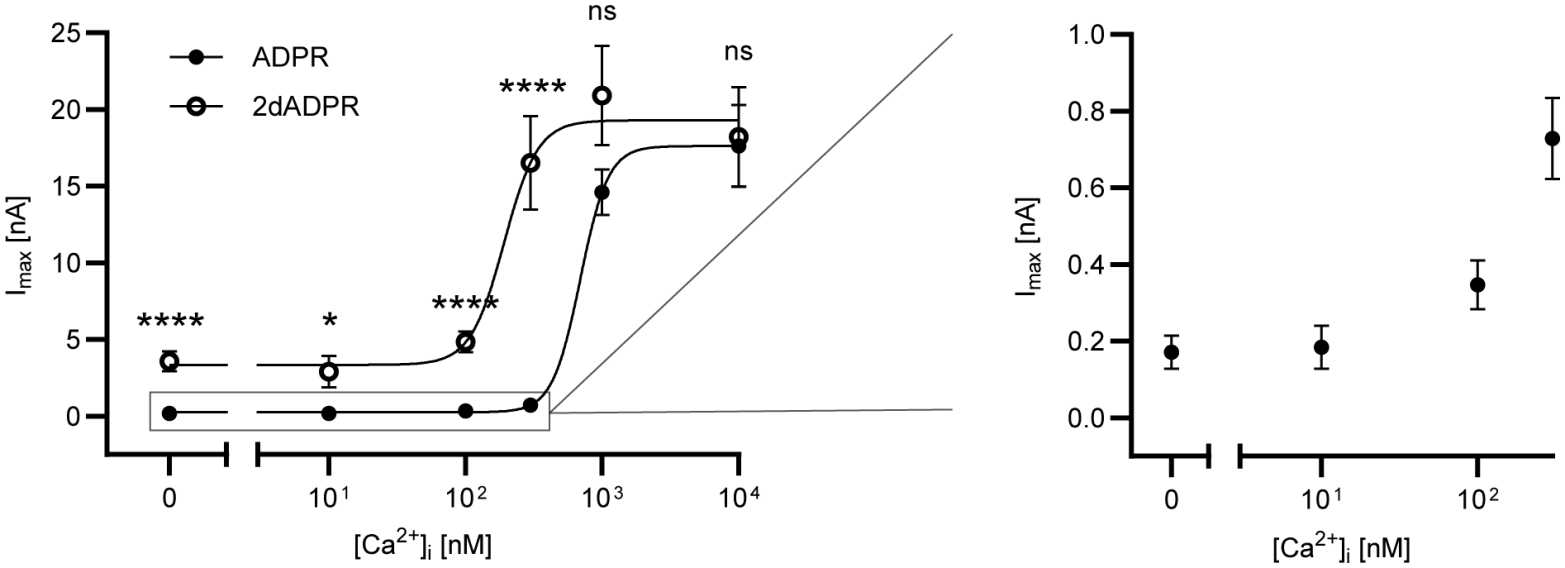
Whole-cell patch clamp experiments were performed with HEK293 cells stably expressing human TRPM2. The intracellular solutions contained 0, 10, 100, 300, 1000 or 10000 nM Ca^2+^ and 100 µM ADPR/2dADPR. The extracellular solution contained 1 mM Ca^2+^. Concentration-response curves yielded EC_50_ values of 694 ± 115 nM (best-fit value ± SE) for ADPR and 192 ± 62 nM for 2dADPR that differed significantly (p<0.01, extra sum-of-squares F test). Currents at [Ca^2+^]_i_ = 0, 10, 100, 300, 1000 and 10000 nM reached 0.17 ± 0.04 (n=12), 0.18 ± 0.06 (n=9), 0.35 ± 0.06 (n=18), 0.73 ± 0.01 (n=22), 14.6 ± 1.50 (n=8) and 17.6 ± 2.68 (n=10) nA for ADPR and 3.58 ± 0.64 (n=10), 2.89 ± 1.04 (n=6), 4.84 ± 0.67 (n=18), 16.5 ± 3.06 (n=16), 20.9 ± 3.24 (n=8) and 18.2 ± 3.23 (n=12) nA for 2dADPR, respectively. Currents are given as means ± SEM with n determinations. ns not significant, *p < 0.05, ****p < 0.0001 (multiple t test).

To assess the Ca^2+^ sensitivity of the activation of hsTRPM2 by 2dADPR as compared to ADPR, we performed whole-cell patch clamp experiments. We used HEK293 cells stably expressing hsTRPM2 and analyzed the maximum whole-cell current induced by 100 µM 2dADPR or ADPR at intracellular Ca^2+^ concentrations ranging from “0 nM” to 10 µM. To obtain a “zero-Ca^2+^” solution, only EGTA but no Ca^2+^ was added to the pipette solution. The extracellular solution contained 1 mM Ca^2+^. Resulting concentration-response curves reveal that activation by 2dADPR has an approx. 4-fold higher Ca^2+^ sensitivity than activation by ADPR with EC_50_ values of 192 ± 62 nM (best-fit value ± SE) and 694 ± 115 nM, respectively (**Figure 1**). 2dADPR currents are > 10-fold higher than ADPR currents at Ca^2+^ concentrations < 1 µM. Interestingly, even at “0 nM” Ca^2 +^ 2dADPR currents are much higher than ADPR currents (2dADPR: 3.58 ± 0.64 nA (mean ± SEM), ADPR: 0.17 ± 0.04 nA). At Ca^2+^ concentrations ≥ 1 µM 2dADPR and ADPR currents are similar. Thus, 2dADPR acts as a superagonist of hsTRPM2 in the physiological range of intracellular Ca^2+^ concentrations.

### 2.2 Activation of hsTRPM2 by 2dADPR is faster than activation by ADPR

**Figure 2:**
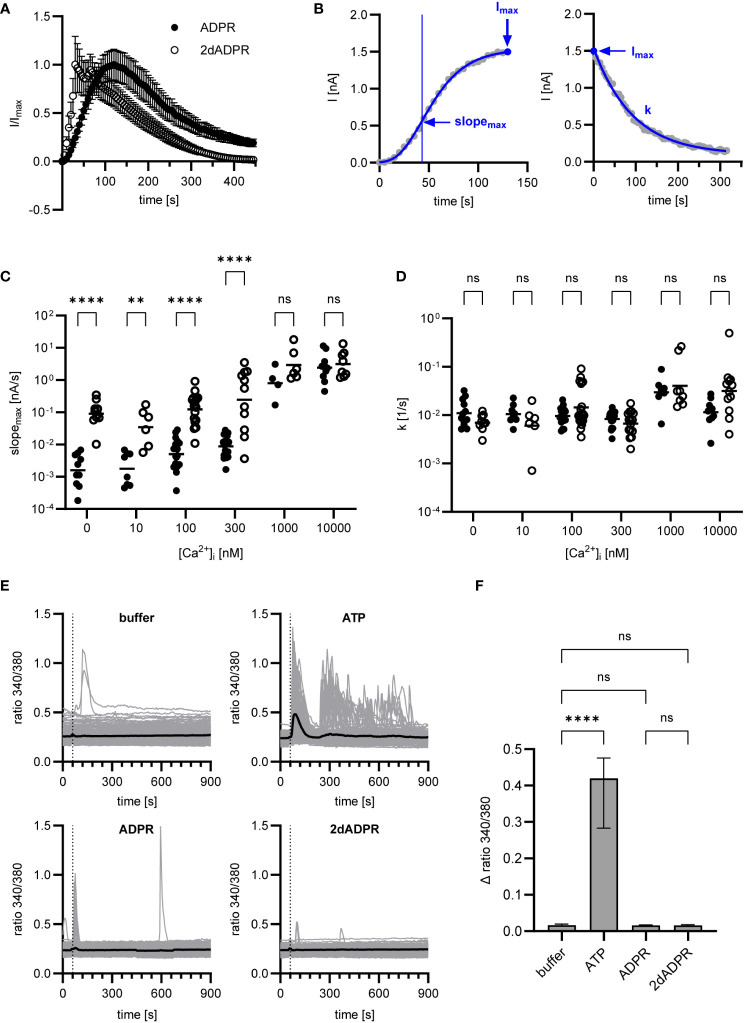
Data of **Figure 1** were analyzed regarding rates of activation and inactivation. **(A)** Time course of normalized whole-cell current over 445 s, exemplarily depicted for [Ca^2+^]_i_ = 300 nM. Currents are given as means ± SEM. **(B)** Rate of activation was determined by fitting the Gompertz model to the development of current over time and calculating the maximum slope of the curve (slope_max_). Rate of inactivation was determined by fitting the exponential decay model to the decline of current over time and employing the exponential decay constant (k). Determination exemplarily depicted for a single measurement at [Ca^2+^]i = 300 nM. Resulting rates of activation **(C)** and inactivation **(D)** for ADPR and 2dADPR. Data displayed log-normal distribution and were log-transformed to obtain normal distribution. They are presented on an anti-log scale. The bar denotes the mean. ns not significant, **p < 0.01, ****p < 0.0001 (multiple t test). **(E)** Ca^2+^ imaging experiments were performed with HEK293 cells stably expressing human TRPM2. 100 µM ATP/ADPR/2dADPR were extracellularly added 1 min after the start of the measurement (dashed line). Negative controls were left without addition (buffer). The bold line represents the mean Fura-2 fluorescence ratio of n=240 (buffer), n=173 (ATP), n=198 (ADPR) and n=166 (2dADPR) cells from 9-10 cell fields from 4 independent experiments. **(F)** The change in ratio from minimum to maximum within the first 2 min after addition was analyzed for each cell. Data displayed non-normal distribution. The bar denotes the median ± 95% CI. ns not significant, **** p < 0.0001 (Kruskal-Wallis test).

To understand how 2dADPR induces higher maximum whole-cell currents at intracellular Ca^2+^ concentrations < 1 µM, we analyzed our data with regard to how fast the current develops and decays again. **Figure 2A**; **Figure S1** show the time course of normalized and unnormalized whole-cell current over 445 s, respectively. As a measure for the rate of activation, the maximum slope of the curve was used after fitting the Gompertz model to the development of current over time (**Figure 2B**, left). For the rate of inactivation, the exponential decay constant after fitting the exponential decay model to the decline of current over time was employed (**Figure 2B**, right). For both agonists, whole-cell current develops faster with increasing intracellular Ca^2+^ (**Figure 2C**). Comparison of rates of activation reveals that activation by 2dADPR is significantly faster than activation by ADPR at all Ca^2+^ concentrations, except for 1 µM and 10 µM. This correlates with significantly higher maximum 2dADPR currents at all Ca^2+^ concentrations, except for 1 µM and 10 µM (compare **Figure 2C** with **Figure 1**). For both agonists, rates of inactivation do not change with increasing intracellular Ca^2+^, showing that, under our experimental conditions, rate of inactivation is independent of Ca^2+^ (**Figure 2D**). Furthermore, the rates of inactivation do not significantly differ between 2dADPR and ADPR. We obtained similar results in patch clamp experiments in which Ca^2+^ was omitted from the extracellular solution. The intracellular solution contained 300 nM Ca^2+^. Whereas maximum whole-cell currents are overall lower, 2dADPR currents are still > 10-fold higher than ADPR currents and activation by 2dADPR is still faster (**Figure S2**). Rates of inactivation still do not differ. This indicates that, while providing positive feedback, extracellular Ca^2+^ does not alter hsTRPM2 activation by 2dADPR in relation to activation by ADPR.

There are reports that in HEK293 cells extracellular ADPR causes IP_3_-dependent Ca^2+^ release from intracellular stores via activation of P2Y receptors (33). Similar observations were made for insulinoma and primary β-cells and the effect of ADPR was attributed to activation of the P2Y1 receptor (34). In our whole-cell patch clamp experiments, HEK293 cells are exposed to ADPR and 2dADPR from the extracellular side when the patch pipette containing intracellular solution approaches the cell. To exclude that Ca^2+^ signals induced by activation of purinergic receptors affect our patch clamp experiments, we performed Ca^2+^ imaging with the HEK293 cells stably expressing hsTRPM2, adding either ATP, ADPR or 2dADPR to the bath. Whereas the cells responded to the established P2Y agonist ATP, neither ADPR nor 2dADPR induced prominent increases in the Fura-2 ratio (**Figures 2E, F**). This indicates that higher TRPM2 currents and faster channel activation are indeed due to 2dADPR acting on hsTRPM2 itself and not caused by activation of purinergic receptors.

### 2.3 Mutation of N-terminal IQ-like motif abrogates hsTRPM2 activation by ADPR and 2dADPR

The Calmodulin Target Database (35, 36) lists over 350 sequences that are known to bind calmodulin. One of these sequences is the so-called IQ-like motif with the pattern (I/F/L/V)Qxxx(R/K)xxxxx. In 2006 Tong et al. (25) discovered an IQ-like motif (aa 406-416) in the N-terminus of hsTRPM2. Using gel shift assays the authors showed that Ca^2+^-bound calmodulin binds to a peptide containing the wild type IQ-like motif (IQDIVRRRQLL), but does not bind to peptides containing mutated motifs in which the characteristic residues are substituted with alanine (AADIVAAAQLA and AADIVRRRQLL). In HEK293 cells transfected with mutated hsTRPM2 (AADIVAAAQLA) the intracellular Ca^2+^ signal after stimulation was completely abolished as compared to cells transfected with wild type hsTRPM2. The authors therefore proposed a mechanism by which Ca^2+^-bound calmodulin binds to the N-terminal IQ-like motif during hsTRPM2 activation.

**Figure 3:**
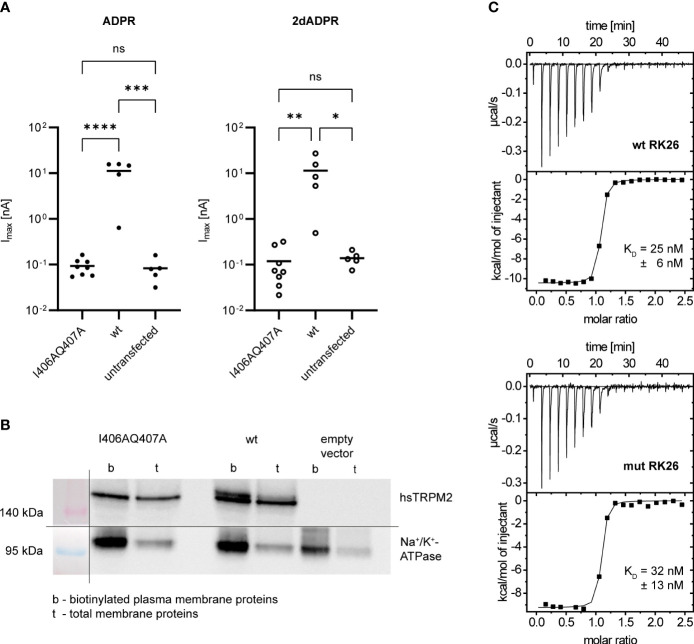
**(A)** Whole-cell patch clamp experiments were performed 24 h after HEK293 cells were transiently transfected with an expression vector containing mutated (I406AQ407A) or wild type (wt) human TRPM2. For negative controls cells were left untransfected. The intracellular solution contained 10 µM Ca^2+^ and 100 µM ADPR/2dADPR. The extracellular solution contained 1 mM Ca^2+^. Data are presented on a log-scale. The bar denotes the mean. ns not significant, *p < 0.05, **p < 0.01, ***p < 0.001, ****p < 0.0001 (one-way ANOVA). **(B)** Expression of human TRPM2 (hsTRPM2) in the plasma membrane confirmed by a cell surface biotinylation assay. It was performed 48 h after HEK293 cells were transiently transfected with an expression vector containing mutated (I406AQ407A) or wild type (wt) or no (empty vector) hsTRPM2. The membrane for Western Blotting was cut in between the 140 kDa and 95 kDa marker band to separately treat the membrane parts with either anti-hsTRPM2 or anti-Na^+^/K^+^-ATPase primary antibody. The shown image represents a grouped image of the two membrane parts. Only detected bands are shown, not full scans of the membrane parts. **(C)** Binding of Ca^2+^-Calmodulin to peptides containing the wild type (wt RK26) or mutated (I406AQ407A, mut RK26) N-terminal IQ-like motif confirmed by isothermal titration calorimetry (ITC). K_D_ values are given as means ± SEM of n = 3 experiments.

Based on the data by Tong et al. (25), we wondered if the N-terminal IQ-like motif indeed plays a role in the Ca^2+^ sensitivity of hsTRPM2 and especially in the difference between 2dADPR and ADPR. We hypothesized that 2dADPR might be able to activate the mutated channel while ADPR is not. To this end, we transiently transfected HEK293 cells with either wild type (wt) or mutated (I406AQ407A) hsTRPM2. We chose the I406AQ407A mutation (equivalent to the AADIVRRRQLL mutated peptide by Tong et al. (25)) because functional data with this mutation is not available so far. We performed whole-cell patch clamp experiments and analyzed the maximum current evoked by 100 µM 2dADPR or ADPR, 10 µM intracellular Ca^2+^ and 1 mM extracellular Ca^2+^. As expected, channel activation by ADPR is abrogated in mutated hsTRPM2 (**Figure 3A**). Similarly, the mutated channel is not activated by 2dADPR. We excluded the possibility that the mutation results in diminished expression of hsTRPM2 in the plasma membrane by performing a cell surface biotinylation assay (**Figure 3B**). The fact that mutated hsTRPM2 is completely inactive, even under high Ca^2+^ conditions, raises the question, if the mutation interferes with the nucleotide dependent activation of hsTRPM2. The observation that mutation of a calmodulin binding site can lead to nonfunctional channels was reported also for TRPV5/6, but it was unclear whether the loss of calmodulin binding was actually the cause for the loss of function (37). We checked if our mutation indeed hinders binding of calmodulin (as stated by Tong et al. (25)) because in this mutation only part of the characteristic residues of an IQ-like motif are substituted with alanine. Isothermal titration calorimetry (ITC) experiments revealed that Ca^2+^-bound calmodulin binds to a peptide containing the wild type IQ-like motif (wt RK26), but does also bind to a peptide containing the I406AQ407A mutated motif (mut RK26), even with similar affinity (**Figure 3C**). These observations suggest that the mutation of the N-terminal IQ-like motif in hsTRPM2 affects channel function independent of calmodulin binding.

### 2.4 Mutation of C-terminal calmodulin binding site results in slower activation of hsTRPM2 by 2dADPR

Alongside the IQ-like motif, the Calmodulin Target Database (35, 36) also lists the so-called 1-14 motif with the pattern (F/I/L/V/W)xxxxxxxxxxxx(F/I/L/V/W). In 2018 we discovered such a calmodulin binding motif (aa 1355-1368) in the C-terminal NUDT9H domain of hsTRPM2 (31). We demonstrated that Ca^2+^-bound calmodulin binds to a peptide containing the wild type motif. However, binding of Ca^2+^-calmodulin to the full-length wild type NUDT9H domain only occurred at 37°C, not at room temperature. We therefore concluded that binding of Ca^2+^-calmodulin to the NUDT9H domain contributes to the temperature sensitivity of hsTRPM2 activation. Surprisingly, we observed that, at room temperature, maximum 2dADPR and ADPR whole-cell currents are similar in HEK293 cells transfected with mutated hsTRPM2 (W1355AI1368A) as compared to cells transfected with wild type hsTRPM2. Since binding of calmodulin does not play a role at room temperature, the cause for this convergence of currents remained unclear.

**Figure 4:**
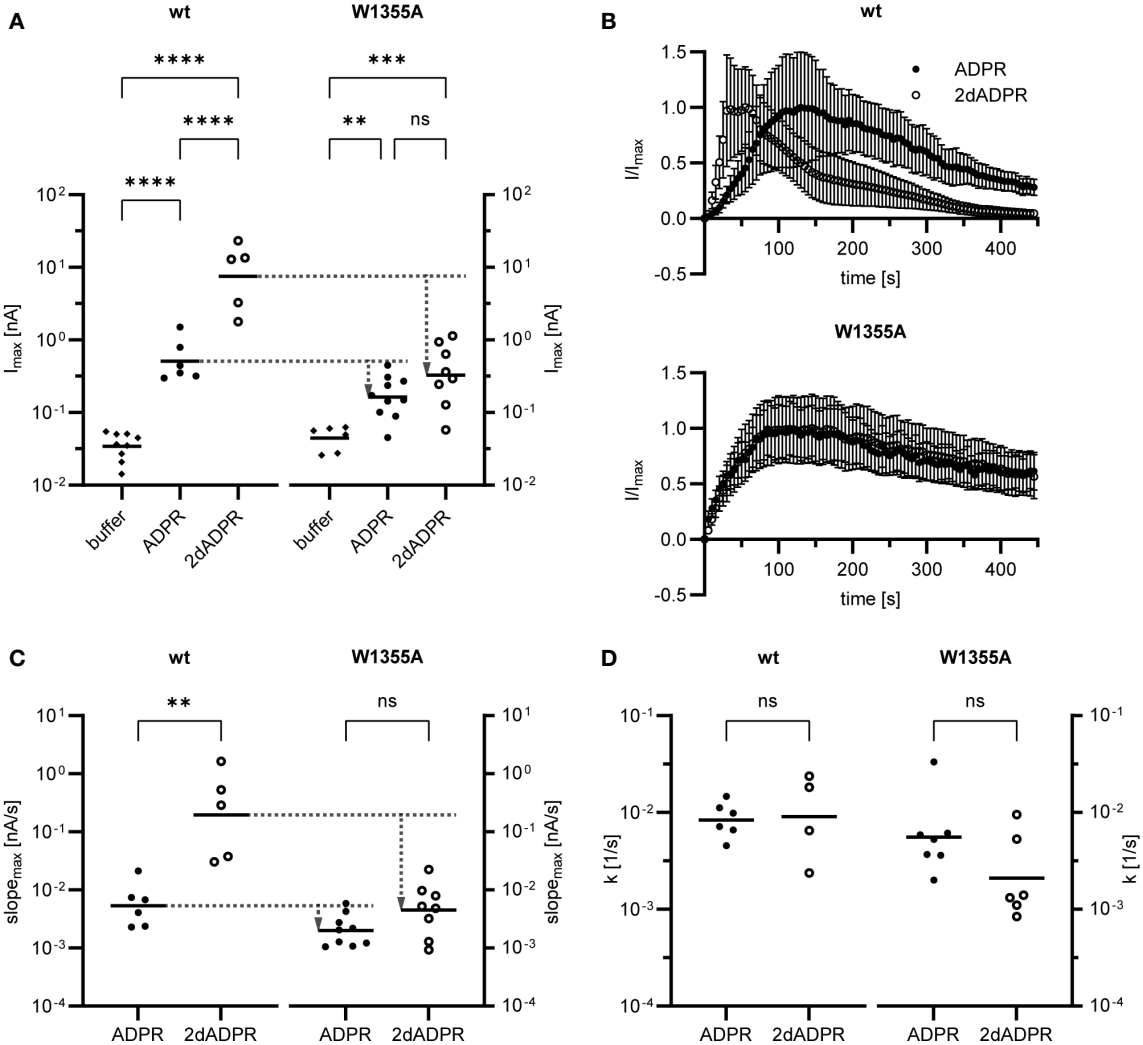
Whole-cell patch clamp experiments were performed 24 h after HEK293 cells were transiently transfected with an expression vector containing mutated (W1355A) or wild type (wt) human TRPM2. The intracellular solution contained 300 nM Ca^2+^ and 100 µM ADPR/2dADPR. For negative controls no agonist was added (buffer). The extracellular solution contained 1 mM Ca^2+^. **(A)** Resulting maximum whole-cell currents. **(B)** Time course of normalized whole-cell current over 445 s. Currents are given as means ± SEM. Rates of activation **(C)** and inactivation **(D)** were determined as described in **Figure 2B**. Data in **(A, C, D)** displayed log-normal distribution and were log-transformed to obtain normal distribution. They are presented on an anti-log scale. The bar denotes the mean. ns not significant, **p < 0.01, ***p < 0.001, ****p < 0.0001 (one-way ANOVA, t-test).

Our results demonstrate that, at intracellular Ca^2+^ concentrations < 1 µM, activation of hsTRPM2 by 2dADPR is faster than activation by ADPR (cf. section 2.2). Since in the W1355AI1368A mutant 2dADPR and ADPR whole-cell currents are similar, we hypothesized that due to this mutation the difference in activation kinetics is absent. We further wondered if mutation of one of the two residues is enough to cause a convergence of currents. To answer these questions, we transiently transfected HEK293 cells with either wild type (wt) or mutated hsTRPM2 in which only the tryptophan is substituted with alanine (W1355A). We performed whole-cell patch clamp experiments and analyzed the maximum current induced by 100 µM 2dADPR or ADPR, 300 nM intracellular Ca^2+^ and 1 mM extracellular Ca^2+^. At this intracellular Ca^2+^ concentration, maximum 2dADPR and ADPR currents are similar in mutated hsTRPM2, whereas 2dADPR currents are expectedly > 10-fold higher than ADPR currents in wild type hsTRPM2 (**Figure 4A**). For both agonists, whole-cell currents are lower in mutated hsTRPM2. However, the convergence of currents in mutated hsTRPM2 results from a relatively higher reduction in 2dADPR current. Compared to wild type hsTRPM2, 2dADPR current is reduced by 96%, whereas ADPR current is reduced by only 68%. **Figure 4B**; **Figure S3** show the time course of normalized and unnormalized whole-cell current over 445 s, respectively. Rates of activation and inactivation were determined from these curves as described in section 2.2. Like whole-cell currents, rates of activation for 2dADPR and ADPR are indeed also similar in mutated hsTRPM2, as opposed to wild type hsTRPM2 (**Figure 4C**). Again, for both agonists, rates of activation are lower in mutated hsTRPM2. However, the convergence of rates of activation in mutated hsTRPM2 results from a relatively higher reduction in the rate of activation for 2dADPR. Compared to wild type hsTRPM2, the rate of activation for 2dADPR is reduced by 98%, whereas the rate of activation for ADPR is reduced by only 62%. In both wild type and mutated hsTRPM2, rates of inactivation for 2dADPR and ADPR are similar (**Figure 4D**). These results demonstrate that 2dADPR induces higher whole-cell currents than ADPR through faster channel activation. We were further able to pinpoint this effect to a single amino acid residue.

## 3 Discussion

When Fliegert et al. (30) discovered 2dADPR as a superagonist of hsTRPM2, experiments at a limited number of intracellular Ca^2+^ concentrations suggested that activation of the channel by 2dADPR has a different Ca^2+^ sensitivity than its activation by ADPR. Here, we demonstrate an approx. 4-fold higher Ca^2+^ sensitivity for 2dADPR as compared to ADPR. Moreover, we show for the first time that 2dADPR can substantially activate human TRPM2 at 0 nM of intracellular Ca^2+^. 2dADPR evoked currents at [Ca^2+^]_i_ = 0 nM may already be high enough to change bulk cytosolic Ca^2+^ and thus induce a global Ca^2+^ signal. This means that stimulation of human TRPM2 by 2dADPR may lead to an activation of resting cells. Currents induced by ADPR appear to be too small to do so. They only reach considerable heights at intracellular Ca^2+^ concentrations that are typically found in already activated cells ([Ca^2+^]_i_ ≥ 300 nM). Stimulation of human TRPM2 by ADPR may therefore play a role in further promoting an already existing activation. These considerations support the notion that 2dADPR activates human TRPM2 in physiological contexts, whereas ADPR does so in pathological pathways (e.g. Ca^2+^ induced cell death (2)). Fliegert et al. (30) originally came to a similar conclusion because they found a biosynthetic pathway for 2dADPR that proceeds via intermediary production of 2′-deoxy-NAD rather than NAD and thus does not interfere with cellular energy metabolism.

We demonstrate that 2dADPR induces higher currents than ADPR at physiological intracellular Ca^2+^ concentrations and that, mechanistically, this is due to faster activation by 2dADPR. Faster activation of whole-cell current likely reflects a higher opening rate of single channels and consequently a higher open probability. In single-channel recordings Fliegert et al. (30) indeed observed that 2dADPR induces a higher open probability than ADPR. However, the authors further reported that inactivation after activation by 2dADPR is slower compared to inactivation after activation by ADPR. Here, we also observed differences in the rate of inactivation between 2dADPR and ADPR, but these differences were only small, as compared to differences in the rate of activation, and statistically not significant. This implies that, at least in the whole-cell configuration, faster activation is the predominant feature of 2dADPR. It was shown that intracellular Ca^2+^ increases the opening rate and open probability of single channels (18). Our data indicate that 2dADPR can “replace” these effects of intracellular Ca^2+^ and thus can independently activate hsTRPM2 at [Ca^2+^]_i_ = 0 nM.

There are repeated reports that hsTRPM2 is regulated by calmodulin (17, 25, 26, 31). However, our data raise doubts as to whether the N-terminal IQ-like motif is involved in this regulation. If not through interfering with calmodulin binding, how then would mutation of this calmodulin binding site affect channel function? The cryo-EM structures of hsTRPM2 (14, 15) provide an alternative explanation: The N-terminal IQ-like motif (aa 406-416) lies within the MHR2 domain and partly interacts with the NUDT9H domain (aa 1235-1503) of the same TRPM2 subunit (14). Q407, for example, form hydrogen bonds with N1259 and E1260. The mutation Q407A that we introduced into full-length hsTRPM2 could therefore disrupt this intra-subunit interaction (also called “interface I” (14)). Consequently, the concerted movement of the MHR1/2 and NUDT9H domain upon TRPM2 activation may not work properly. Interestingly, a similar link becomes apparent for the C-terminal calmodulin binding site (aa 1355-1368): It lies within the P-loop of the NUDT9H domain which closely interacts with the MHR1/2 domain of the adjacent TRPM2 subunit (14, 15). In the closed state of the channel, it interacts with the MHR1 domain (“interface III” (14)/”resting interface” (15)). Upon binding of ADPR, this interface is disrupted and a new interface with the MHR2 domain is formed (“activation interface” (15)). The transition between the resting and activation interface is enabled by a rigid-body rotation of the NUDT9H core region toward the NUDT9H cap region and subsequent domain closure. Thereby, the NUDT9H core region is pulled away from the MHR1 domain, which in turn promotes closure of the MHR1/2 domain. These motions of the intracellular nucleotide binding domains are the main driving force for channel opening (15). Our data indicate that binding of 2dADPR induces conformational changes that differ from those induced by ADPR in such a way that transition from the resting to the activation interface and thus channel opening is facilitated. Residue W1355 seems to play a crucial role therein, since mutation of this residue neutralizes the higher rate of channel activation induced by 2dADPR as compared to ADPR. Binding of Ca^2+^ to its binding site in the transmembrane domain of hsTRPM2 is currently viewed as an additional requirement for channel opening (15). However, the finding that 2dADPR strongly activates hsTRPM2 in the absence of intracellular Ca^2+^ indicates that the conformational changes induced by binding of 2dADPR promote channel opening so much that initial binding of Ca^2+^ is not as required as it is for hsTRPM2 activation by ADPR. Such a mode of hsTRPM2 activation has not been described so far. Structures of hsTRPM2 in complex with 2dADPR, especially those in the absence of Ca^2+^, are of high interest and would further clarify this issue.

In conclusion, we demonstrate that 2dADPR induces higher hsTRPM2 currents than ADPR by means of faster channel activation. The difference in activation kinetics is neutralized by mutation of a single amino acid residue (W1355) in the NUDT9H domain of hsTRPM2. Ultimately, activation of hsTRPM2 by 2dADPR has a higher Ca^2+^ sensitivity than activation by ADPR, rendering even lower than resting intracellular Ca^2+^ concentrations sufficient to substantially activate human TRPM2.

## 4 Materials and methods

### 4.1 Cell culture and transfection vectors

HEK293 wild type cells and HEK293 cells stably expressing hsTRPM2 (clone #24) were used for experiments. The generation of the stable cell line has been described previously (38). The cells were cultivated in DMEM (with GlutaMAX-I and 4.5 g/l glucose, Thermo Fisher Scientific) supplemented with 10% FBS (Merck) and 100 U/ml penicillin-100 µg/ml streptomycin (Thermo Fisher Scientific). T25 flasks were kept at 37°C and 5% CO_2_. 400 µg/ml of the selective antibiotic G418 (Merck) was added to the medium of HEK293 #24 cells.

HEK293 wild type cells were transiently transfected with an expression vector for hsTRPM2 (pIRES2-EGFP-hsTRPM2), the generation of which has been described previously (39). Mutations were inserted using the QuickChange Site-Directed Mutagenesis Kit (Agilent Technologies). The coding sequences were confirmed by DNA sequencing (Eurofins Genomics).

### 4.2 Solutions for patch clamp

The extracellular solution for whole-cell patch clamp experiments contained (in mM): 140 *N*-methyl-d-glucamine (NMDG), 5 KCl, 3.3 MgCl_2_, 1 CaCl_2_, 5 glucose and 10 HEPES (pH = 7.4, adjusted with HCl at room temperature). Since NMDG does not permeate through hsTRPM2, the replacement of Na^+^ by NMDG^+^ in the extracellular solution prevents large inward currents that can lead to cell rupture during the recording. For the extracellular solution without Ca^2+^, no CaCl_2_ was added (nominal Ca^2+^ free). The intracellular solutions contained (in mM): 8 NaCl, 120 KCl, 1 MgCl_2_ and 10 HEPES (pH = 7.2, adjusted with KOH at room temperature). Free [Ca^2+^] in the intracellular solutions was adjusted to 0, 10, 100, 300, and 1000 nM with 10 mM EGTA and 0, 0.54, 3.62, 6.30 and 8.50 mM CaCl_2_, respectively. For the intracellular solution with 10 µM free [Ca^2+^] a combination of EGTA and nitrilotriacetic acid (NTA) was used (40). NTA has a low selectivity for Ca^2+^ over Mg^2+^ and thus free [Mg^2+^] was controlled for. 2.86 mM EGTA, 7.14 mM NTA, 3.05 mM CaCl_2_ and 3.28 mM MgCl_2_ were used to obtain 10 µM free [Ca^2+^] and 1 mM free [Mg^2+^]. All necessary metal concentrations were calculated with WEBMAXC (41). Free [Ca^2+^] in the intracellular solutions was verified by fluorimetric [Ca^2+^] measurements. As calcium indicator Fura-2 (K_D_ = 236 nM (42), Merck) was chosen for intracellular solutions with free [Ca^2+^] ≤ 1 µM and Fura-2FF (K_D_ = 6 µM (43), ATT Bioquest) for the intracellular solution with 10 µM free [Ca^2+^].

### 4.3 Electrophysiology (patch clamp)

HEK293 cells stably expressing hsTRPM2 were seeded to 35 mm dishes and incubated for 24 h at 37°C. HEK293 wild type cells were transiently transfected with a pIRES2-EGFP expression vector containing mutated (I406AQ407A/W1355A) or wild type (wt) hsTRPM2 or were left untransfected. 2.5 µg vector DNA were mixed with 5 µl jetPEI reagent (Polyplus Transfection), both dissolved in 125 µl 150 mM NaCl solution (Polyplus Transfection). After 30 min incubation at room temperature the jetPEI/DNA complexes (250 µl total) were added to 2.5*10^5^ cells suspended in 1 ml medium. The cells and jetPEI/DNA complexes were then distributed to 35 mm dishes and incubated for 24 h at 37°C.

Patch clamp experiments were performed with an EPC 10 Patch Clamp Amplifier (HEKA) and the PATCHMASTER data acquisition software (v2x90.2, HEKA) at room temperature. The medium in the 35 mm dishes was replaced by 1 ml of extracellular solution. 100 µM ADPR (Merck) or 2dADPR (Biolog Life Sciences) were added to the respective intracellular solution from 20 mM stock solutions. For negative controls no agonist was added. Pipettes were pulled from 1.05*1.50*80 mm glass capillaries (Science Products) with a P-97 Micropipette Puller (Sutter Instrument) and filled with intracellular solution. After pipette resistance (1-3 MΩ) was determined, a target cell was chosen and the expression of hsTRPM2 confirmed by fluorescence microscopy. Whole-cell configuration was established by formation of a gigaseal and subsequent breaking of the membrane patch. The series resistance compensation was set to 70%. Macroscopic current was recorded by setting the holding potential to -50 mV and repetitively applying voltage ramps of 160 ms spanning the range from -85 mV to +20 mV every 5 s over a recording period of 445 s. For data analysis, outward current at +15 mV over the entire recording time was drawn from each experiment.

Nonlinear regression (least squares regression) of patch clamp data was performed with Prism (v9.5.1, GraphPad Software). To obtain concentration-response curves, the four-parameter logistic (4PL) model was fit to maximum outward currents (I_max_) at different intracellular Ca^2+^ concentrations. To determine rate of activation, the Gompertz growth model (y = y_M_*(y_0_/y_M_)^(exp(-k*x))) was fit to the development of current from the start of the recording to the maximum current reached. Subsequently, the slope of the curve at the point of inflection (slope_max_) was calculated with MATLAB (v9.12, MathWorks). To determine rate of inactivation, the model for one phase exponential decay was fit to the decay of current from the maximum current reached to the end of the recording and the rate constant of exponential decay (k) was used.

### 4.4 Biotinylation of cell surface proteins

HEK293 wild type cells, grown in T25 flasks to 50-60% confluency, were transiently transfected with a pIRES2-EGFP expression vector containing mutated (I406AQ407A), wild type (wt) or no (empty vector) hsTRPM2 using Lipofectamine LTX and Plus Reagent (Thermo Fisher Scientific). The cells were incubated for 48 h at 37°C and successful transfection was confirmed by fluorescence microscopy. To biotinylate plasma membrane proteins, the cells were washed and treated with 1 mg/ml EZ-Link Sulfo-NHS-LC-Biotin (Thermo Fisher Scientific). Cells were then collected by centrifugation and washed. Cell lysis and isolation of membrane proteins was performed with the ProteoExtract Native Membrane Protein Extraction Kit (Merck). To separate biotinylated plasma membrane proteins from all (total) membrane proteins, 300 µg of membrane proteins were mixed with 50 µl NeutrAvidin Agarose Resin Beads (Thermo Fisher Scientific) and incubated overnight rotating at 4°C. The beads were collected by centrifugation afterwards. Gels for the SDS-PAGE were casted with the Mini-Protean Casting System (Bio-Rad Laboratories) and contained 7.5% acrylamide in the resolving gel and 3.9% in the stacking gel. The beads and 10 µg of total membrane proteins were mixed with sample buffer, heated at 75°C for 7 min, centrifuged and loaded to the gel. In case of the bead sample only the supernatant (biotinylated plasma membrane proteins without beads) was used. After the SDS-PAGE (100 V, approx. 2 h) the samples were transferred to an Immobilon-P PVDF Transfer Membrane (Merck) by a Western Blot (0.4 A, approx. 1 h). The membrane was cut in between the 140 kDa and 95 kDa marker band (Spectra Multicolor Broad Range Protein Ladder, Thermo Fisher Scientific). Both membrane parts were washed and blocked for 1 h at room temperature. The upper membrane part was then treated with rabbit anti-hsTRPM2 antibody (1:50000 dilution, NB500-241, Novus Biologicals) overnight at 4°C. The lower membrane part was treated with rabbit anti-Na^+^/K^+^-ATPase antibody (1:1000 dilution, #3010, Cell Signaling Technology) in the same way. Both membrane parts were washed and incubated with an HRP-conjugated goat anti-rabbit antibody (1:10000 dilution, #111-035-045, Dianova) for 1 h at room temperature. For detection a 1:10 mixture of SuperSignal West Dura Extended Duration Substrate and Super Signal West Pico Chemiluminescent Substrate (Thermo Fisher Scientific) was used. Both membrane parts were washed and incubated with the detection solution for 5 min at room temperature. The membranes were then photographed with an exposure time of 4.6 s.

### 4.5 Isothermal titration calorimetry

Peptides had the sequences RIVEWTKKIQDIVRRRQLLTVFREGK (wt RK26) and RIVEWTKKAADIVRRRQLLTVFREGK (mut RK26). They were purchased from GL Biochem and their identity was confirmed by LC-MS.

Recombinant calmodulin was produced using a pET15b expression vector containing the coding sequence for wild type human calmodulin without additional tags. *E. coli* BL21 Gold (DE3) cells (Agilent Technologies) were transformed and grown at 37°C in LB Lennox medium (Carl Roth) supplemented with 100 µg/ml ampicillin (Merck). At an optical density (OD_600_) of 0.6 cells were induced with 100 µM isopropyl β-d-1-thiogalactopyranoside (IPTG, Merck). Protein expression was carried out for 16 h at 20°C. Harvested cells were lyzed by pulsed sonication for 3*3 min (30% power, Bandelin Sonopuls GM2070, Bandelin electronic) and the target protein was purified from the cleared lysate by hydrophobic interaction chromatography (HIC) on a HiTrap Phenyl FF (LS) column (Cytiva). Lysis and loading were performed in 50 mM Tris (pH = 7.5) and 2 mM CaCl_2_, elution was performed in 50 mM Tris (pH = 7.5) and 10 mM EDTA. Elution fractions were pooled and subjected to size exclusion chromatography (SEC) on a Superdex S75 10/300 column (Cytiva) in 20 mM HEPES (pH = 7.5), 150 mM NaCl and 5 mM CaCl_2_. Peak fractions were pooled and protein identity was confirmed by SDS-PAGE and MS.

Isothermal titration calorimetry (ITC) experiments were performed with a MicroCal iTC200 microcalorimeter (Malvern Panalytical) at 25°C. Peptides were dissolved in the SEC buffer used for the purification calmodulin. 200 µM peptide in the syringe were titrated into 16 µM calmodulin in the ITC cell. After an initial injection, 18 injections of 2 µl interspaced by 150 s were added to the cell at a stirring speed of 750 rpm. Peptide into buffer and buffer into protein titrations were used to correct for baseline deviation due to heat of dilution.

Analysis of ITC data was performed with the MicroCal ORIGIN software. One-site binding models were fit to the data and K_D_ values were calculated. They are given as means ± SEM of n = 3 independent experiments.

### 4.6 Ca^2+^ imaging

HEK293 cells stably expressing hsTRPM2 were seeded to 8 well µ-slides (ibidi) at a density of 4*10^4^ cells/well and incubated for 24 h at 37°C. The slides were previously coated with 50 µg/ml human fibronectin (Merck) to allow for cell adherence.

Prior to Ca^2+^ imaging cells were incubated with 4 µM Fura-2/AM (Merck) for 30 min at 37°C. Afterwards cells were washed and covered with 200 µl buffer, which contained (in mM): 140 NaCl, 5 KCl, 1 MgSO_4_, 1 CaCl_2_, 5.5 Glucose, 1 NaH_2_PO_4_ and 20 HEPES (pH = 7.4). The slides were then placed on an inverted fluorescence microscope (DM IRBE, Leica) with a 40x oil immersion objective. Images were acquired every 5 s over 15 min using the Volocity software (v6.6.2, PerkinElmer) with an EM-CCD camera (C9100-13, Hamamatsu). A Lambda DG-4 (Sutter instrument) was used as the light source, a HC 340/26 nm and HC 387/11 nm excitation filter, a 400DCLP beam splitter and a 510/84 nm emission filter (AHF analysentechnik). ATP, ADPR or 2dADPR at a final concentration of 100 µM were added 1 min after the start of the measurement. For negative controls an equal volume of buffer was added to the cells.

For analysis of Ca^2+^ imaging data regions of interest (ROIs) were selected using ImageJ (44) with the StarDist plugin (45). Ratio and Δ ratio values were then calculated with Excel (Microsoft). Cells exhibiting an increased ratio before addition of nucleotide (pre-stimulated cells) were excluded from the analysis.

### 4.7 Statistical analysis

Statistical analysis of the data was performed with Prism (v9.5.1/v10.0.3 GraphPad Software). Data was tested for normal or log-normal distribution with the D’Agostino-Pearson omnibus K2 test. For small n, the Kolmogorov-Smirnov test was used. In case of normal distribution data are reported as means ± SEM and parametric tests (t-test, multiple t-test, one-way ANOVA) were applied including correction for multiple comparisons (Holm-Šidák test, Tukey test). For concentration-response curves, it was tested if the best-fit value of a selected unshared parameter (EC_50_) differs between the two data sets (extra sum-of-squares F test). In case of log-normal distribution data were log-transformed to obtain normal distribution. In case of non-normal distribution data are reported as medians ± 95% CI and non-parametric tests (Kruskal-Wallis test) were applied including correction for multiple comparisons (Dunn’s test). For all tests α = 0.05 was adopted. ns not significant, * p < 0.05, ** p < 0.01, *** p < 0.001, **** p < 0.0001.

## Data availability statement

The original contributions presented in the study are included in the article/**Supplementary Material**. Further inquiries can be directed to the corresponding author.

## Author contributions

JP: Conceptualization, Data curation, Formal analysis, Investigation, Methodology, Visualization, Writing – original draft, Writing – review & editing. SS: Data curation, Formal analysis, Investigation, Methodology, Visualization, Writing – review & editing. SE: Investigation, Writing – review & editing. AR: Formal analysis, Writing – review & editing. HT: Conceptualization, Funding acquisition, Methodology, Resources, Supervision, Writing – review & editing. AG: Conceptualization, Funding acquisition, Resources, Supervision, Writing – review & editing. RF: Conceptualization, Data curation, Formal analysis, Funding acquisition, Methodology, Supervision, Visualization, Writing – review & editing, Resources. 
