# Supplementary material for: 2′-deoxy-ADPR activates human TRPM2 faster than ADPR and thereby induces higher currents at physiological Ca2+ concentrations
Source: Front Immunol. 2024 Jan 22;15:1294357. doi: 10.3389/fimmu.2024.1294357 (PMC10838996; doi:10.3389/fimmu.2024.1294357)
Supplement: Supplementary file 1 [file DataSheet_1.docx]

Supplementary Material


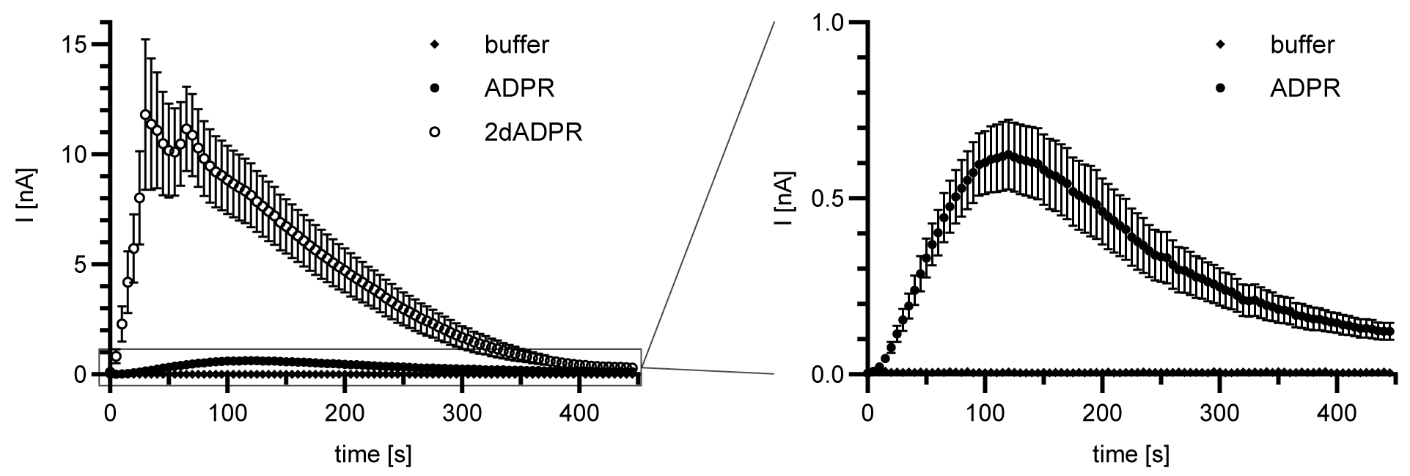


**Supplementary Figure 1.** Whole-cell patch clamp experiments were performed with HEK293 cells stably expressing human TRPM2. The intracellular solutions contained 0, 10, 100, 300, 1000 or 10000 nM Ca^2+^ and 100 μM ADPR/2dADPR. For negative controls no agonist was added (buffer). The extracellular solution contained 1 mM Ca^2+^. The time course of whole-cell current over 445 s is exemplarily depicted for [Ca^2+^]_i_ = 300 nM. Currents are given as means ± SEM of n=6 (buffer), n=22 (ADPR) and n=16 (2dADPR) experiments.


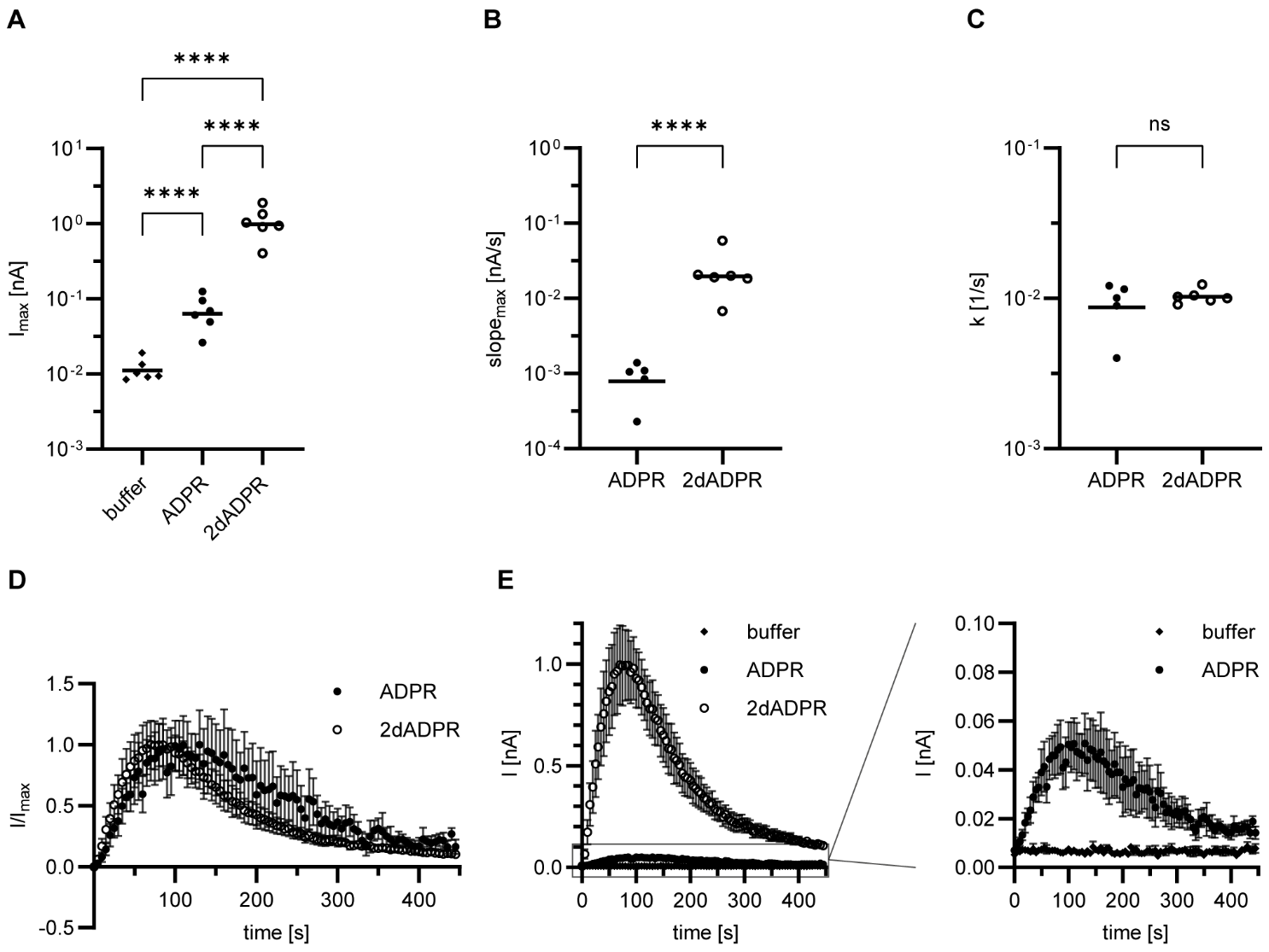


**Supplementary Figure 2.** Whole-cell patch clamp experiments were performed with HEK293 cells stably expressing human TRPM2. The intracellular solution contained 300 nM Ca^2+^ and 100 μM ADPR/2dADPR. For negative controls no agonist was added (buffer). Ca^2+^ was omitted from the extracellular solution (nominal Ca^2+^ free). **(A)** Resulting maximum whole-cell currents. Rates of activation **(B)** and inactivation **(C)** were determined as described in Fig. 2B. Data in (A), (B) and (C) displayed log-normal distribution and were log-transformed to obtain normal distribution. They are presented on an anti-log scale. The bar denotes the mean. ns not significant, **** p < 0.0001 (one-way ANOVA, t-test). **(D)** Time course of normalized and unnormalized **(E)** whole-cell current over 445 s. Currents are given as means ± SEM.


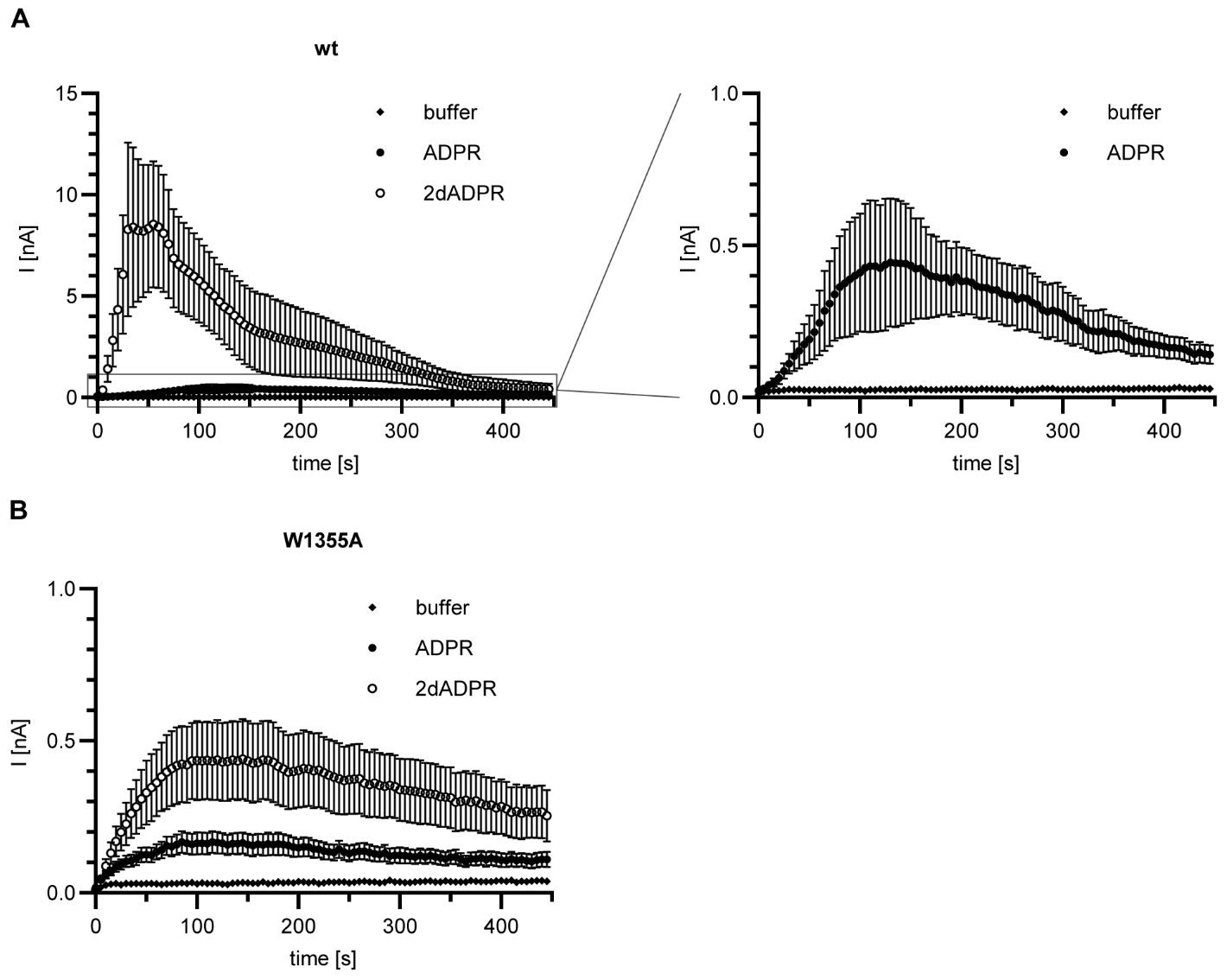


**Supplementary Figure 3.** Whole-cell patch clamp experiments were performed 24 h after HEK293 cells were transiently transfected with an expression vector containing mutated (W1355A) or wild type (wt) human TRPM2. The intracellular solution contained 300 nM Ca^2+^ and 100 μM ADPR/2dADPR. For negative controls no agonist was added (buffer). The extracellular solution contained 1 mM Ca^2+^. Depicted is the time course of whole-cell current over 445 s for wt **(A)** and mutated (W1355A) **(B)** TRPM2. Currents are given as means ± SEM of n=9 (buffer), n=6 (ADPR) and n=5 (2dADPR) experiments for wt and n=6 (buffer), n=10 (ADPR) and n=8 (2dADPR) experiments for mutated (W1355A) TRPM2.
